# Turtles of the genera *Geoemyda* and *Pangshura* (Testudines: Geoemydidae) lack differentiated sex chromosomes: the end of a 40-year error cascade for *Pangshura*

**DOI:** 10.7717/peerj.6241

**Published:** 2019-02-06

**Authors:** Sofia Mazzoleni, Barbora Augstenová, Lorenzo Clemente, Markus Auer, Uwe Fritz, Peter Praschag, Tomáš Protiva, Petr Velenský, Lukáš Kratochvíl, Michail Rovatsos

**Affiliations:** 1 Department of Ecology, Charles University, Prague, Czech Republic; 2 Museum of Zoology, Senckenberg, Dresden, Germany; 3 Turtle Island, Graz, Austria; 4 landsnails.org, Prague, Czech Republic; 5 Prague Zoological Garden, Prague, Czech Republic

**Keywords:** Comparative genome hybridization, FISH, Sex determination, Evolution, Telomeres, Microsatellite, Karyotype, Turtles, Sex chromosomes

## Abstract

For a long time, turtles of the family Geoemydidae have been considered exceptional because representatives of this family were thought to possess a wide variety of sex determination systems. In the present study, we cytogenetically studied *Geoemyda spengleri* and *G. japonica* and re-examined the putative presence of sex chromosomes in *Pangshura smithii*. Karyotypes were examined by assessing the occurrence of constitutive heterochromatin, by comparative genome hybridization and *in situ* hybridization with repetitive motifs, which are often accumulated on differentiated sex chromosomes in reptiles. We found similar karyotypes, similar distributions of constitutive heterochromatin and a similar topology of tested repetitive motifs for all three species. We did not detect differentiated sex chromosomes in any of the species. For *P. smithii*, a ZZ/ZW sex determination system, with differentiated sex chromosomes, was described more than 40 years ago, but this finding has never been re-examined and was cited in all reviews of sex determination in reptiles. Here, we show that the identification of sex chromosomes in the original report was based on the erroneous pairing of chromosomes in the karyogram, causing over decades an error cascade regarding the inferences derived from the putative existence of female heterogamety in geoemydid turtles.

## Introduction

Turtles exhibit different sex determination modes. Although it is still a matter of debate, the ancestral (Valenzuela & Adams, 2011; Johnson Pokorná & Kratochvíl, 2016) and most common sex determination mechanism in turtles is most likely environmental sex determination (ESD). Genotypic sex determination (GSD) evolved independently in five families (Chelidae, Emydidae, Geoemydidae, Kinosternidae, Trionychidae) (Valenzuela & Adams, 2011; Badenhorst et al., 2013). Turtles of the family Geoemydidae (Old World pond turtles) are a fascinating model for the evolution of sex determination because it has been reported that this large family with more than 70 species (Rhodin et al., 2017) includes lineages with ESD as well as GSD, with both male (XX/XY sex chromosomes) and female (ZZ/ZW) heterogamety (Valenzuela & Adams, 2011).

Environmental sex determination was reported for three geoemydid genera, namely *Mauremys* (including *Chinemys*) (Rhodin et al., 2017), *Melanochelys* and *Rhinoclemmys*, mainly based on skewed sex ratios of hatchlings incubated at different temperatures (Ewert, Etchberger & Nelson, 2004). So far, cytogenetic examinations revealed XX/XY sex chromosomes only in the black marsh turtle *Siebenrockiella crassicollis* (Carr & Bickham, 1986; Kawagoshi, Nishida & Matsuda, 2012) and ZZ/ZW sex chromosomes only in the brown roofed turtle *Pangshura smithii* (Sharma, Kaur & Nakhasi, 1975). The XX/XY sex chromosomes of *S. crassicollis* are medium-sized and have been assigned as the fourth pair of the karyogram. The sex chromosomes are heteromorphic and with gene content partially homologous to chromosome pair five of chicken (*Gallus gallu*s) and Chinese softshell turtle (*Pelodiscus sinensis*) (Kawagoshi, Nishida & Matsuda, 2012). The Y chromosome is metacentric, and the X chromosome is submetacentric, with a prominent C-positive band, missing on the Y. Despite that the X and Y chromosomes differ in morphology and C-banding pattern, it seems that they share gene content extensively. Sex-specific regions were not detected after single-copy gene mapping (Kawagoshi, Nishida & Matsuda, 2012). Therefore, we assume that these sex chromosomes are at an early stage of differentiation with a small sex-specific region. For *P. smithii*, ZZ/ZW sex chromosomes have been reported by Sharma, Kaur & Nakhasi (1975) based on distinct chromosome morphology. For the majority of species of the family Geoemydidae, the sex determination mode remains unstudied.

In the current investigation, we cytogenetically explored the brown roofed turtle *P. smithii*, the black-breasted leaf turtle *Geoemyda spengleri* and the Ryukyu black-breasted leaf turtle *G. japonica*. The genus *Geoemyda* is especially interesting because it represents the sister taxon of *Siebenrockiella*, the genus with evident male heterogametic sex chromosomes (Carr & Bickham, 1986; Kawagoshi, Nishida & Matsuda, 2012). In addition, the genus *Geoemyda* is phylogenetically nested in a major geoemydid clade containing also *Pangshura* (Spinks et al., 2004; Lourenço et al., 2013; Pereira et al., 2017), a genus with reported female heterogametic sex chromosomes (Sharma, Kaur & Nakhasi, 1975).

The pioneering studies by Nakamura (1937, 1949) reported a chromosome number of 2*n* = 52 for *G. spengleri*, but karyotypes were not documented photographically. In addition, neither the sex of the examined turtles nor their geographical origin was reported. We assume that Nakamura (1937, 1949) actually studied *G. japonica*, since the Japanese populations had the status of a subspecies of *G. spengleri* at that time (Yasukawa, Ota & Hikida, 1992). Chaowen, Ming & Liuwang (1998) studied later undoubtedly *G. spengleri*, using individuals originating from Hunan Province, China. Chaowen, Ming & Liuwang (1998) applied classic cytogenetic methods and revealed also a karyotype of 2*n* = 52 chromosomes. However, no other cytogenetic approach has been applied to *Geoemyda* yet. Besides the karyogram of *P. smithii* published by Sharma, Kaur & Nakhasi (1975), no further cytogenetic studies exist for this species.

In the present study, we constructed karyograms for all three species and further explored their karyotypes by C-banding stain to reveal the distribution of constitutive heterochromatin. Furthermore, we examined the presence of differentiated sex chromosomes by comparative genome hybridization (CGH) and fluorescence *in situ* hybridization with repetitive elements that often accumulate on sex chromosomes of reptiles, such as telomeric motifs, (GATA)_8_ microsatellite repeats and rDNA loci (Literman, Badenhorst & Valenzuela, 2014; Matsubara et al., 2016; Rovatsos et al., 2017a; Augstenová et al., 2018).

## Materials and Methods

### Samples and species verification

Blood samples from four individuals of *G. japonica*, five individuals of *G. spengleri* and four individuals of *P. smithii* (Table 1) were used for preparation of mitotic chromosome suspensions and DNA isolation. All turtles are captive-bred or legally imported, and kept in Zoo Plzeň (Czech Republic), Prague Zoo (Czech Republic), or the Museum of Zoology, Senckenberg Dresden (Germany).

**Table 1:** Number of individuals per species and sex, analyzed in this study.

Species	Sex	
	♂	♀
*Geoemyda japonica*	2	2
*Geoemyda spengleri*	3	2
*Pangshura smithii*	2	2

Genomic DNA was extracted using a DNeasy Blood and Tissue Kit (Qiagen, Venlo, Netherlands). We amplified by PCR and sequenced the mitochondrial cytochrome *b* gene (cyt *b*), in order to verify the taxon and to provide a DNA-based identity of our cytogenetically examined material for future comparison (for the same approach see Koubová et al., 2014; Rovatsos et al., 2015a, 2016a; Rovatsos, Johnson Pokorná & Kratochvíl, 2015b). Cyt *b* was amplified by PCR using the primers L14919 5′-AACCACGGTTGTTATTCAACT-3′ and H16064 5′-CTTTGGTTTACAAGAACAATGCTTTA-3′ (Burbrink, Lawson & Slowinski, 2000; De Queiroz, Lawson & Lemos-Espinal, 2002). The PCR reaction protocol consists of 20–80 ng of DNA, one μl of each primer (10 pmol/μl), five μl of 10× PCR buffer (Bioline GmbH, Luckenwalde, Germany), 2.5 μl of MgCl_2_ (50 mM), one μl of dNTPs (10 mM each), 0.5 μl of BioTaq DNA polymerase (5 U/μl, Bioline) and water up to final volume of 50 μl. The amplification conditions were: 95 °C for 3 min, followed by 35 cycles of 95 °C for 30 s, 50 °C for 30 s, and 72 °C for 1 min, and the final step of 72 °C for 5 min. The PCR products were sequenced by Macrogen (Seoul, South Korea), and the obtained sequences deposited in GenBank. A BLAST search (Altschul et al., 1990) was performed to compare our sequences with those previously deposited in public databases.

### Chromosome preparation and staining

Mitotic chromosome suspensions were prepared from all studied individuals using whole blood cell cultures. For leukocyte cultivation, 100–300 μl of blood samples were cultured at 30 °C for a week without CO_2_ supplementation in 5 ml of DMEM medium (Gibco) enriched with 10% fetal bovine serum (Gibco, Carlsbad, CA, USA), 100 μg/ml lipopolysacharide (Sigma-Aldrich, St. Louis, MO, USA), 2 mM L-glutamine (Sigma-Aldrich, St. Louis, MO, USA), 3% phytohaemaglutinin M solution (Gibco), 100 units/ml of penicillin and 100 μg/ml of streptomycin (Gibco). Three hours before harvesting, 35 μl of colchemid solution (10 μg/ml stock solution, Roche, Basel, Switzerland) was added to the medium. Chromosome suspensions were obtained according to the standard method, including an initial hypotonic treatment with 0.075M KCl at 37 °C for 30 min and four times fixation in 3:1 methanol/acetic acid solution. Chromosome suspensions were stored in a freezer for further use.

Chromosomal spreads were stained with Giemsa solution, and selected metaphases were captured in a Provis AX70 (Olympus Corporation, Tokyo, Japan) fluorescence microscope, equipped with a DP30BW digital camera (Olympus). Subsequently, karyograms were constructed using Ikaros karyotyping software (Metasystems, Altlussheim, Germany).

The distribution of constitutive heterochromatin was detected by C-banding (Sumner, 1972). The slides were aged at 55 °C for 1 h, then soaked successively in 0.2N HCl at room temperature for 45 min, in 5% Ba(OH)_2_ solution at 45 °C for 4–5 min and in 2xSSC for 1 h at 60 °C, with intermediate washes in distilled water, and finally stained with 4′,6-diamidino-2-phenylindole (DAPI) and mounted with antifade medium Vectashield (Vector Laboratories, Burlingame, CA, USA).

### Fluorescence *in situ* hybridization with probes for repetitive elements

The probe to detect the topology of rDNA loci was prepared from a plasmid (pDm r.a51#1) with an 11.5-kb insertion, encoding the 18S and 28S rRNA units of *Drosophila melanogaster* (Endow, 1982) and labelled with biotin-dUTP using a Nick Translation Kit (Abbott Laboratories, Chicago, IL, USA).

The probe for telomeric motifs (TTAGGG)_*n*_ was produced and labelled with biotin-dUTP in a single PCR reaction using the primers (TTAGGG)_5_ and (CCCTAA)_5_ without a DNA template (Ijdo et al., 1991). The probes for the detection of rDNA loci and telomeric motifs were ethanol-precipitated with sonicated salmon sperm DNA and subsequently resuspended in hybridization buffer (50% formamide/2xSSC) (Rovatsos et al., 2015a, 2017b).

The probe for the GATA microsatellite motif was synthesized by Macrogen (Seoul, South Korea) as (GATA)_8_ and labelled with biotin. Subsequently, 0.3 μl of (GATA)_8_ biotin-labelled probe (100 pmol/μl stock solution) was diluted in 10 μl of hybridization buffer (50% formamide, 20xSSC, 10% sodium dodecyl sulphate, 10% dextran sulphate, 1× Denhard’s buffer, pH = 7) per slide.

The preparation of chromosome spreads and probes, the hybridization conditions, the post-hybridization washes, the signal amplification and detection are explained in detail in Rovatsos et al. (2015a). At least 20 metaphases per slide were captured to confirm the fluorescent signal. The pictures were collected in black and white and superimposed with colors. The photos were processed with DP Manager imaging software (Olympus).

### Comparative genome hybridization

To detect putative sex-specific chromosome regions, CGH was used according to our standard protocol (Rovatsos et al., 2015a). In each species, equal amounts of male and female genomic DNA (one μg each) were labelled independently with biotin-dUTP and digoxigenin-dUTP, respectively, using a Nick translation kit (Abbott Laboratories, Chicago, IL, USA) and then mixed together. Sonicated salmon sperm DNA was added and ethanol-precipitation was carried out overnight at −20 °C. The labelled DNA was resuspended in hybridization buffer, denatured at 75 °C for 10 min and immediately chilled on ice for 10 min prior to hybridization. The slides with chromosomal material were subsequently treated with RNase A and pepsin, fixed with 1% formaldehyde, dehydrated through an ethanol series, denatured in 70% formamide/2xSSC at 75 °C for 3 min, dehydrated again and air-dried. Hybridization was performed at 37 °C for 2 or 3 days. Post-hybridization washes were performed three times in 50% formamide/2xSSC at 42 °C for 5 min and twice in 2xSSC at room temperature for 5 min. Afterward, the slides were incubated in 100 μl of 4xSSC/5% blocking reagent (Roche, Basel, Switzerland) at 37 °C for 30 min and then with 100 μl of 4xSSC/5% blocking reagent including avidin-FITC (Vector Laboratories) and anti-digoxigenin rhodamine (Roche, Basel, Switzerland) at 37 °C for 30 min. The slides were washed in 4xSSC/0.05% Tween 20, dehydrated, air dried, stained with DAPI, and mounted with Vectashield (Vector Laboratories).

## Results

### Species verification

The mitochondrial cyt *b* gene was successfully amplified by PCR and sequenced in all three examined species. A BLAST search (Altschul et al., 1990) of the obtained sequences verified the expected taxonomic identity of the turtles examined here as *G. japonica*, *G. spengleri*, and *P. smithii*. The haplotypes are deposited in GenBank, under the accession numbers MK097237–MK097240.

### Karyotype reconstruction and C-banding

**Figure 1:**
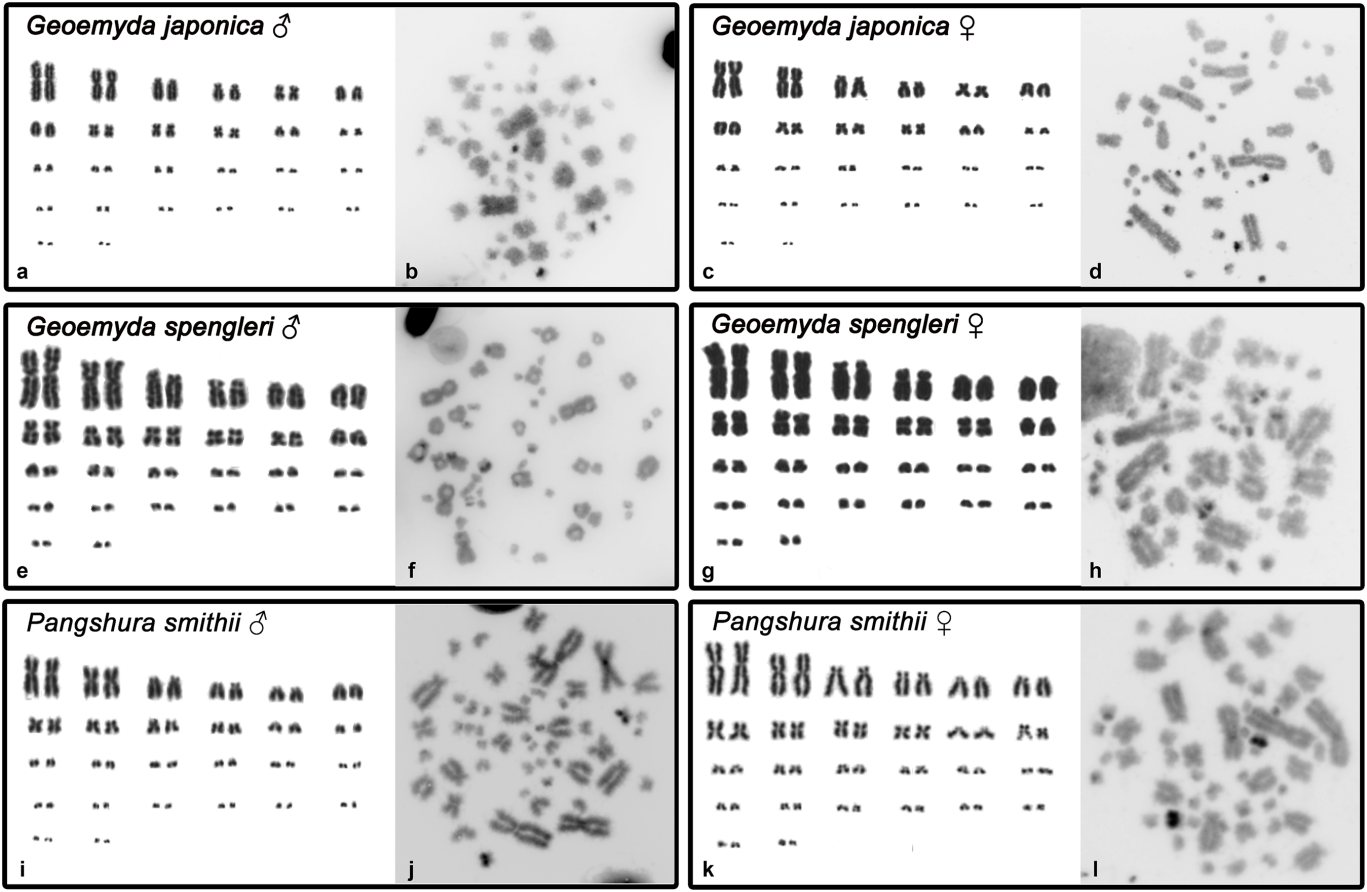
Karyograms and C-banded metaphases of *Geoemyda japonica* (A–D), *Geoemyda spengleri* (E–H), and *Pangshura smithii* (I–L). Please note that microchromosomes are paired according to size for illustration, which does not correspond to actual homology of chromosomes.

Both *G. japonica* and *G. spengleri* have a similar karyotype with 2*n* = 52 chromosomes composed of 12 pairs of macrochromosomes, gradually decreasing in size, and 14 pairs of microchromosomes. Among macrochromosomes, nine pairs are bi-armed and three are acrocentric (pairs 6, 7, and 11) (Fig. 1). C-positive bands were identified in the centromeric regions of almost all chromosomes. A prominent heterochromatic block has been detected in the chromosome pair 12 in metaphases of both sexes in both species (Fig. 1).

In addition, *P. smithii* has a similar karyotype with 2*n* = 52, consisting of 12 pairs of bi-armed macrochromosomes and 14 pairs of microchromosomes. C-positive heterochromatin was detected in the centromeric regions of all chromosomes. An extensive accumulation of constitutive heterochromatin was detected in pair 12 in both sexes. This chromosome pair seems to be polymorphic in size in some individuals, but this polymorphism is not linked to sex.

### Fluorescence *in situ* hybridization and comparative genome hybridization

**Figure 2:**
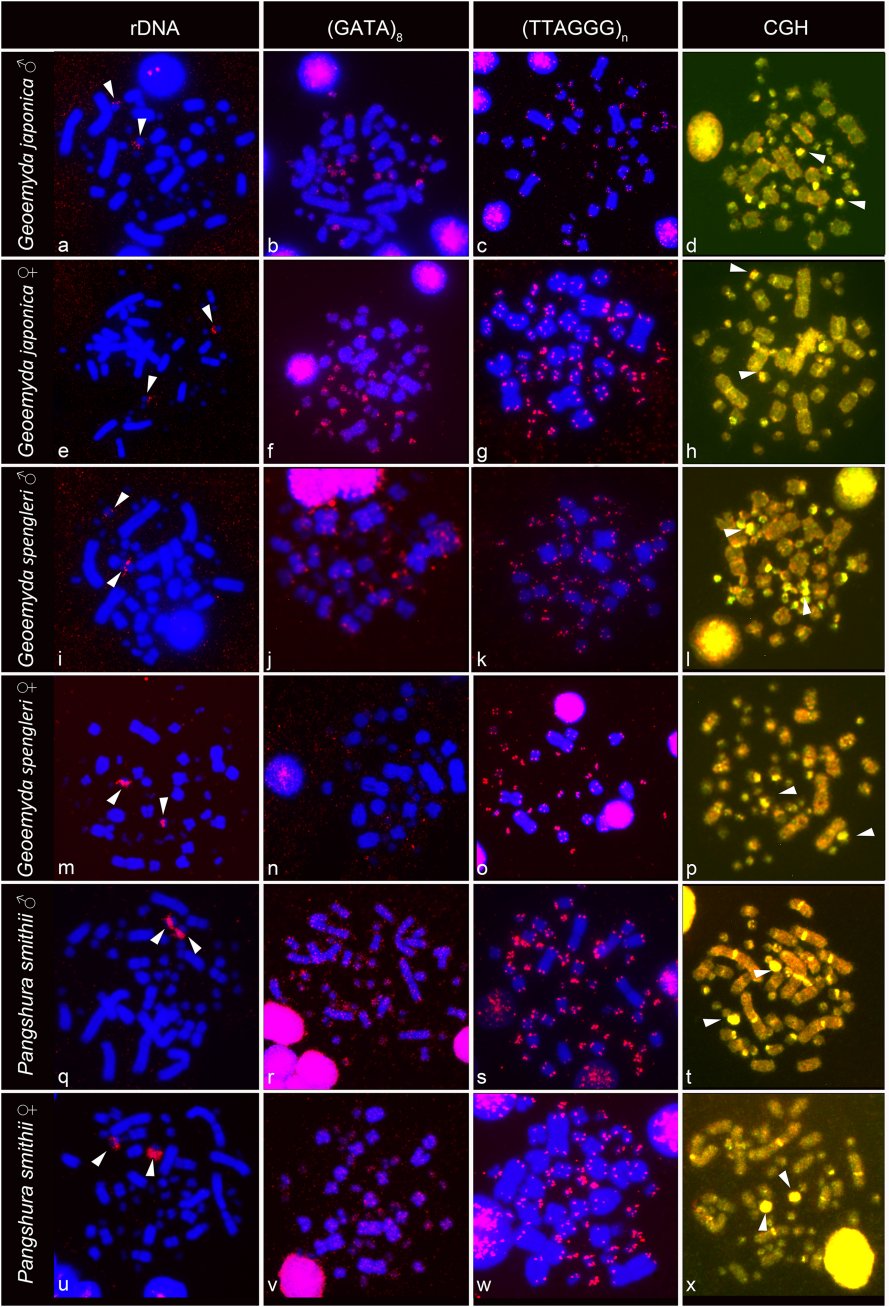
FISH with rDNA, (GATA)_8_ and telomeric probes in metaphases of *Geoemyda japonica* (A–H), *Geoemyda spengleri* (I–P), and *Pangshura smithii* (Q–X). Chromosomes are stained blue with DAPI, and the signal of the probe is pseudocolored in red. In CGH, the male genome is stained with FITC (green color) and the female genome with rhodamine (red color). Genomic regions common for both sexes appear yellow due to the combination of green and red color. Chromosomal regions with similar sequence content in both sexes are visualized in yellow. Arrows indicate the chromosome pair 12, with the prominent C-positive block.

The rDNA loci are located in the terminal position in a pair of microchromosomes in *G. japonica* and *P. smithii*, and near the centromere in a pair of microchromosomes in *G. spengleri* (Fig. 2). The rDNA loci seem to be in all three species linked to the chromosome pair 12, which has the prominent C-positive heterochromatic block.

The telomeric repeats (TTAGGG)_*n*_ showed the expected terminal chromosome topology in all studied individuals (Fig. 2). The (GATA)_8_ microsatellite motif had a widespread distribution in several pairs of microchromosomes and in the centromeric region of a pair of acrocentric macrochromosomes in both species of the genus *Geoemyda* but without any sex-specific signal. A weak signal of the (GATA)_8_ microsatellite motif was detected in the telomeric regions of several chromosomes in *P. smithii* but without any sex-specific pattern. CGH did not reveal any sex-specific differences in any of the three species (Fig. 2).

## Discussion

Our results confirmed that all three studied species have similar karyotypes with 2*n* = 52 chromosomes, which agrees with former studies (Nakamura, 1949; Sharma, Kaur & Nakhasi, 1975; Killebrew, 1977; Yasukawa, Ota & Hikida, 1992; Chaowen, Ming & Liuwang, 1998). *Geoemyda* is phylogenetically close to two lineages (*Pangshura*, *Siebenrockiella*) (Spinks et al., 2004; Lourenço et al., 2013; Pereira et al., 2017) for which differentiated sex chromosomes have been reported (Carr & Bickham, 1986; Sharma, Kaur & Nakhasi, 1975; Kawagoshi, Nishida & Matsuda, 2012). In several non-avian reptiles, differentiated sex chromosomes are often highly conserved across the phylogenetic spectrum, for example in trionychid turtles (Rovatsos et al., 2017b), lacertids (Rovatsos et al., 2016b), iguanas (Rovatsos et al., 2014), and caenophidian snakes (Rovatsos et al., 2015c). However, our cytogenetic analysis using multiple approaches did not reveal any differentiated sex chromosomes in *G. spengleri* and *G. japonica*. Thus, turtles of this genus have either GSD with poorly differentiated sex chromosomes not detectable by our cytogenetic techniques or ESD where sex chromosomes are lacking (following the definition of ESD by Johnson Pokorná & Kratochvíl (2016)).

**Figure 3:**
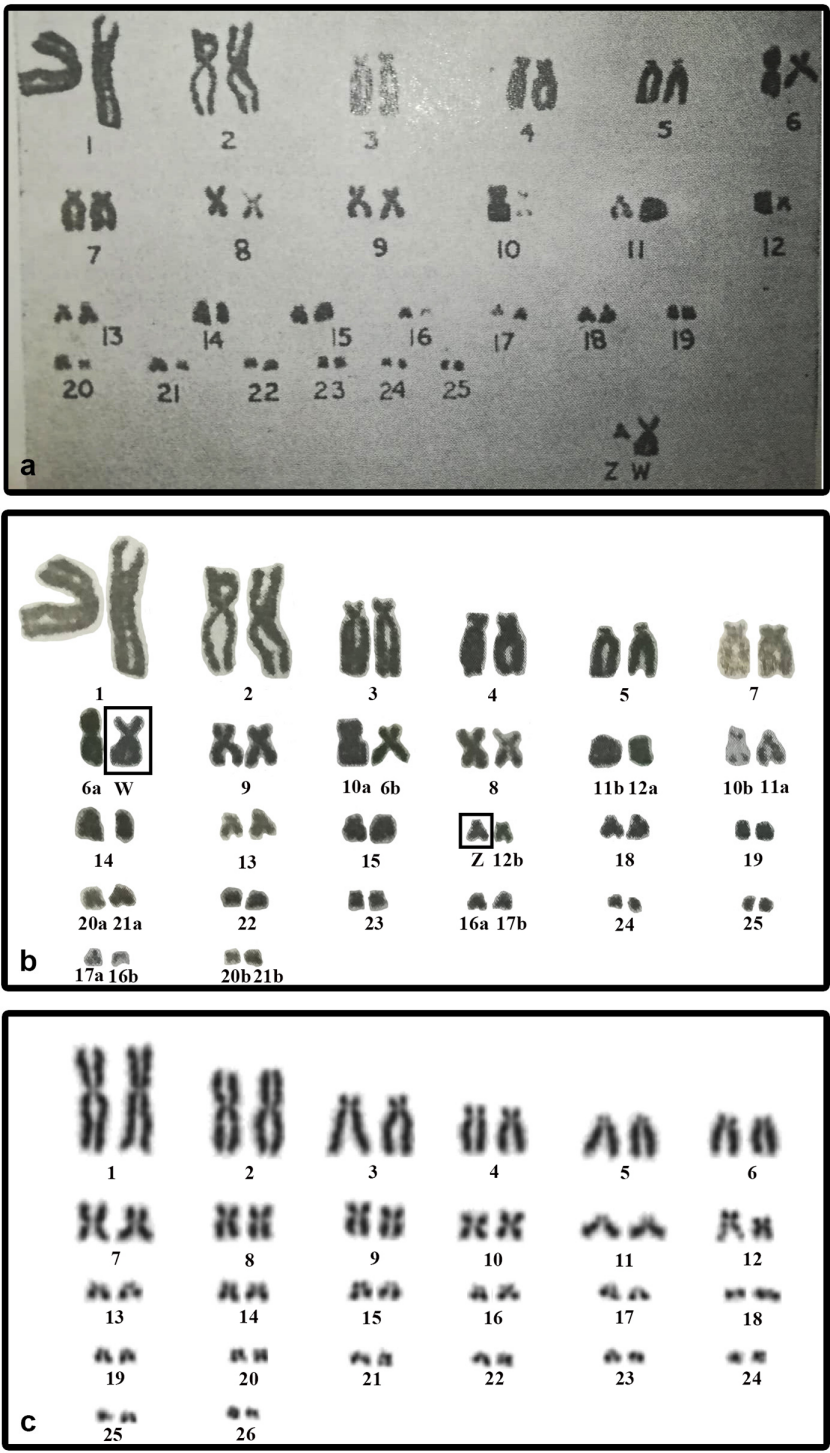
The original karyogram of Sharma, Kaur & Nakhasi (1975) (A), their karyogram re-arranged by us (B), and a new karyogram of a female individual from our studied material (C). Note that the chromosomes misidentified as Z and W in the original study (A) can be autosomal and easily assigned according to size and morphology into the pairs 16–26 and 7–9, respectively, in our new karyogram (C). Numbers in the re-arranged karyogram (B) refer to the original assignment of chromosome pairs by Sharma, Kaur & Nakhasi (1975).

We did not detect sex chromosomes in *P. smithii* despite differentiated, highly heteromorphic ZZ/ZW sex chromosomes had been shown by Sharma, Kaur & Nakhasi (1975) in a karyogram of this species based on Giemsa-stained metaphase chromosomes. In this study, the Z chromosome of *P. smithii* was identified as a small acrocentric chromosome, while the W chromosome was shown as a medium-sized metacentric chromosome. To explain the discrepancies between our results and those of Sharma, Kaur & Nakhasi (1975), we revisited their karyogram (Fig. 3A) and we discovered several potential errors in their assignment of chromosomes to homologue pairs that likely contributed to the mischaracterization of *P. smithii* as possessing a ZZ/ZW system (Fig. 3B). Namely, the chromosome identified by Sharma, Kaur & Nakhasi (1975) as the Z chromosome is a microchromosome, and we conclude that it can be better reassigned as a homolog of one of the pairs 16–26. Additionally, the metacentric chromosome identified by Sharma, Kaur & Nakhasi (1975) as the W chromosome could be reassigned as a homolog of pair 7, 8, or 9. After simple rearrangement of the original karyogram, no obviously heteromorphic pair of chromosomes is detectable (Fig. 3B), consistent with our own karyotyping of new specimens (Fig. 3C).

We found variability in size between the homologous chromosomes in the pair 12 of all three examined species of turtles. This pair includes heterochromatic blocks co-localizing with the accumulation of rDNA repeats (Figs. 1 and 2). Heterochromatic blocks are often connected with autosomal polymorphism due to rapid divergence of repeat numbers (Altmanová et al., 2016), and a polymorphism in chromosome morphology including rDNA genes was reported also in ESD species of geoemydid turtles such as *Rhinoclemmys pulcherrima* (Carr & Bickham, 1986). The polymorphism of the chromosome pair 12 is not linked to sex in *G. spengleri*, *G. japonica*, or *P. smithii*. Thus, there is no evidence that this pair corresponds to sex chromosomes. In any case, the chromosome pair 12 was not identified by Sharma, Kaur & Nakhasi (1975) as sex chromosomes, although it might contribute to the incorrect pairing of chromosomes in their karyotype (Fig. 3).

**Figure 4:**
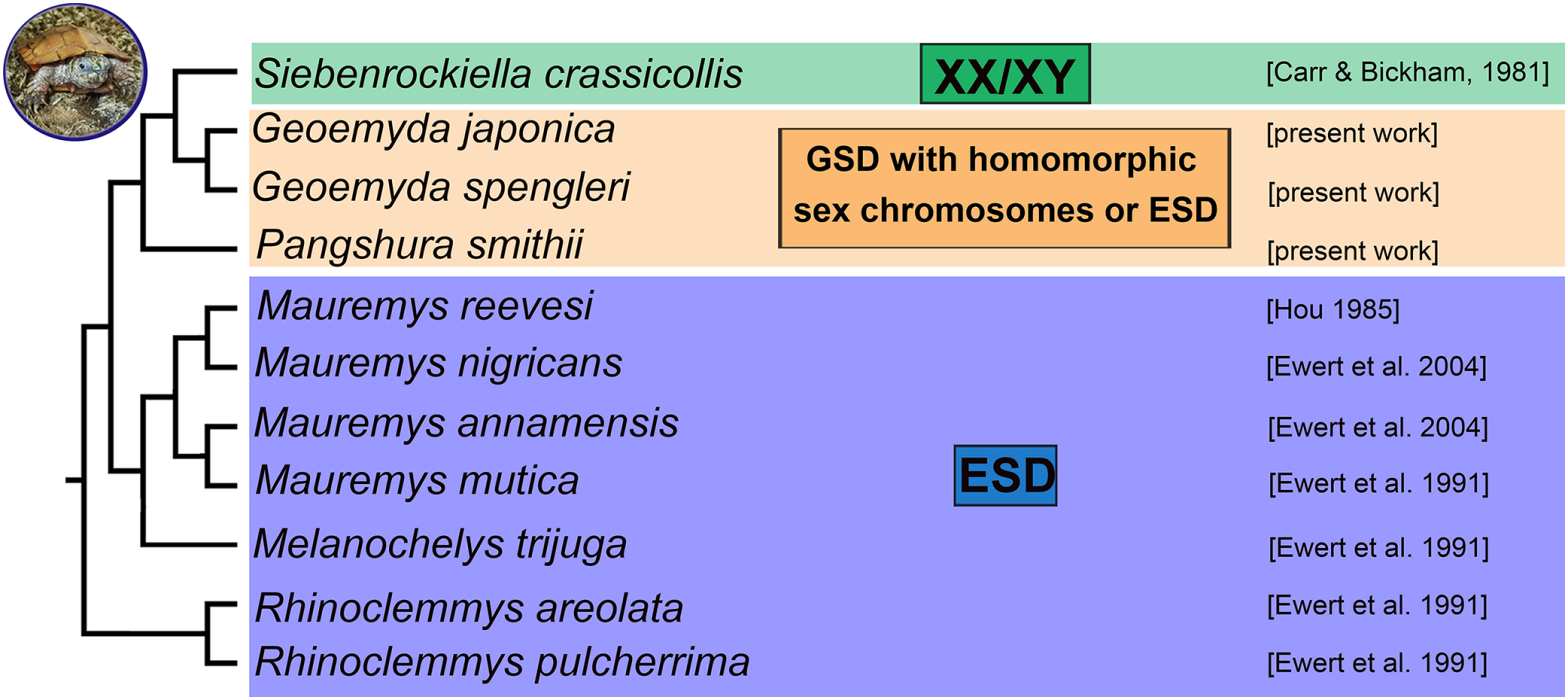
Phylogenetic reconstruction of the sex determination modes in turtles from the family Geoemydidae. Phylogenetic relationships follow Spinks et al. (2004), Lourenço et al. (2013) and Pereira et al. (2017).

According to our results, there is no evidence for female heterogamety with differentiated sex chromosomes in geoemydid turtles of the genus *Pangshura*. Thus, among turtles, female heterogamety is only known in softshell turtles (Trionychidae) (Badenhorst et al., 2013; Rovatsos et al., 2017b). In the family Geoemydidae, the only reliable identification of sex chromosomes refers to the XX/XY sex determination system of *S. crassicollis* (Carr & Bickham, 1981; Kawagoshi, Nishida & Matsuda, 2012), while other studied species possess either ESD as most other lineages of the family Geoemydidae with known sex determination (Fig. 4) or, perhaps, GSD with poorly differentiated and homomorphic sex chromosomes.

Unfortunately, the erroneous identification of putative sex chromosomes in *P. smithii* was influential for scientific literature. It impacted studies examining the cytogenetics of turtles (Martinez et al., 2008; Kawagoshi, Nishida & Matsuda, 2012) and comparative phylogenetic reconstructions as well as reviews of sex determination mechanisms, causing a 40-year error cascade regarding the inferred number of sex chromosome turnovers in amniotes and the evolution of sex determination and genome organization (Modi & Crews, 2005; Gamble, 2010; Valenzuela & Adams, 2011; Badenhorst et al., 2013; Johnson Pokorná & Kratochvíl, 2016; Montiel et al., 2017). The error cascade caused by the putative sex chromosomes of *P. smithii* illustrates how little we still know about sex determination in reptiles and that even traditionally widely accepted reports of sex determination modes can benefit from re-examination with modern molecular cytogenetic methods and broader species sampling.

## Conclusions

We found that *G. spengleri*, *G. japonica*, and *P. smithii* share karyotypes with 2*n* = 52 chromosomes and a similar topology of constitutive heterochromatin and repetitive motifs. We did not detect differentiated sex chromosomes in any of these species. It is particularly notable in *P. smithii*, where a ZZ/ZW sex determination system with differentiated sex chromosomes was described more than 40 years ago. This information was repeated in subsequent reviews and phylogenetic analyses on sex determination in amniotes and influenced their outcomes and conclusions. We show that the identification of sex chromosomes in the original report was based on the erroneous pairing of chromosomes in their karyogram. We conclude that additional research is needed in order to clarify the true sex determination mode in the three studied turtle species, which might possess either GSD with poorly differentiated sex chromosomes not detectable by our cytogenetic techniques or ESD as most other lineages of the family Geoemydidae with known sex determination (Fig. 4). Future research should include controlled incubation experiments of eggs to examine the influence of temperature in hatchling sex ratios in *G. spengleri*, *G. japonica*, and *P. smithii*, as well as molecular cytogenetic examination of additional geoemydid species, to gain a better understanding of the evolution of sex determination in this group.

## Supplemental Information

10.7717/peerj.6241/supp-1 Supplemental Information 1 Haplotype sequences from cyt b gene from the turtles cytogenetically examined in this study.

## Additional Information and Declarations

### Data Availability

The following information was supplied regarding data availability: The raw data is included in the figures, in Genbank (MK097237–MK097240) and in the Supplemental Information.

## References

[ref-1] Altmanová M, Rovatsos M, Kratochvíl L, Johnson Pokorná M (2016). Minute Y chromosomes and karyotype evolution in Madagascan iguanas (Squamata: Iguania: Opluridae). Biological Journal of the Linnean Society.

[ref-2] Altschul SF, Gish W, Miller W, Myers EW, Lipman DJ (1990). Basic local alignment search tool. Journal of Molecular Biology.

[ref-3] Augstenová B, Mazzoleni S, Kratochvíl L, Rovatsos M (2018). Evolutionary dynamics of the W chromosome in caenophidian snakes. Genes.

[ref-4] Badenhorst D, Stanyon R, Engstrom T, Valenzuela N (2013). A ZZ/ZW microchromosome system in the spiny softshell turtle, *Apalone spinifera*, reveals an intriguing sex chromosome conservation in Trionychidae. Chromosome Research.

[ref-5] Burbrink FT, Lawson R, Slowinski JB (2000). Mitochondrial DNA phylogeography of the polytypic North American rat snake (*Elaphe obsoleta*): a critique of the subspecies concept. Evolution.

[ref-40] Carr JL, Bickham JW (1981). Sex chromosomes of the Asian black pond turtle, Siebenrockiella crassicollis (Testudines: Emydidae). Cytogenetics and Cell Genetics.

[ref-6] Carr JL, Bickham JW (1986). Phylogenetic implications of karyotypic variation in the Batagurinae (Testudines: Emydidae). Genetica.

[ref-7] Chaowen G, Ming W, Liuwang N (1998). A cytogenetic study on three species of turtle. Acta Hydrobiologica Sinica.

[ref-8] De Queiroz A, Lawson R, Lemos-Espinal JA (2002). Phylogenetic relationships of North American garter snakes (*Thamnophis*) based on four mitochondrial genes: how much DNA sequence is enough?. Molecular Phylogenetics and Evolution.

[ref-9] Endow SA (1982). Polytenization of the ribosomal genes on the X and Y chromosomes of *Drosophila melanogaster*. Genetics.

[ref-10] Ewert MA, Etchberger CR, Nelson CE, Valenzuela N, Lance VA (2004). Turtle sex-determining modes and TSD patterns, and some TSD pattern correlates. Temperature-Dependent Sex Determination in Vertebrates.

[ref-11] Gamble T (2010). A review of sex determining mechanisms in geckos (Gekkota: Squamata). Sexual Development.

[ref-12] Ijdo JW, Wells RA, Baldini A, Reeders ST (1991). Improved telomere detection using a telomere repeat probe (TTAGGG)_n_ generated by PCR. Nucleic Acids Research.

[ref-13] Johnson Pokorná M, Kratochvíl L (2016). What was the ancestral sex-determining mechanism in amniote vertebrates?. Biological Reviews.

[ref-14] Kawagoshi T, Nishida C, Matsuda Y (2012). The origin and differentiation process of X and Y chromosomes of the black marsh turtle (*Siebenrockiella crassicollis*, Geoemydidae, Testudines). Chromosome Research.

[ref-15] Killebrew F (1977). Mitotic chromosomes of turtles. IV. The Emydidae. Texas Journal of Science.

[ref-16] Koubová M, Johnson Pokorná M, Rovatsos M, Farkačová K, Altmanová M, Kratochvíl L (2014). Sex determination in Madagascar geckos of the genus *Paroedura* (Squamata: Gekkonidae): are differentiated sex chromosomes indeed so evolutionary stable?. Chromosome Research.

[ref-17] Literman R, Badenhorst D, Valenzuela N (2014). qPCR-based molecular sexing by copy number variation in rRNA genes and its utility for sex identification in soft-shell turtles. Methods in Ecology and Evolution.

[ref-18] Lourenço JM, Glémin S, Chiari Y, Galtier N (2013). The determinants of the molecular substitution process in turtles. Journal of Evolutionary Biology.

[ref-19] Martinez PA, Ezaz T, Valenzuela N, Georges A, Graves JAM (2008). An XX/XY heteromorphic sex chromosome system in the Australian chelid turtle *Emydura macquarii*: a new piece in the puzzle of sex chromosome evolution in turtles. Chromosome Research.

[ref-20] Matsubara K, O’Meally D, Azad B, Georges A, Sarre SD, Graves JAM, Matsuda Y, Ezaz T (2016). Amplification of microsatellite repeat motifs is associated with the evolutionary differentiation and heterochromatinization of sex chromosomes in Sauropsida. Chromosoma.

[ref-21] Modi WS, Crews D (2005). Sex chromosomes and sex determination in reptiles. Current Opinion in Genetics & Development.

[ref-22] Montiel EE, Badenhorst D, Tamplin J, Burke RL, Valenzuela N (2017). Discovery of the youngest sex chromosomes reveals first case of convergent co-option of ancestral autosomes in turtles. Chromosoma.

[ref-23] Nakamura K (1937). On the chromosomes of some chelonians (a preliminary note) [in Japanese]. Japanese Journal of Genetics.

[ref-24] Nakamura K (1949). A study in some chelonians with notes on chromosomal formula in the Chelonia [in Japanese]. Chromosome.

[ref-25] Pereira AG, Sterli J, Moreira FR, Schrago CG (2017). Multilocus phylogeny and statistical biogeography clarify the evolutionary history of major lineages of turtles. Molecular Phylogenetics and Evolution.

[ref-26] Rhodin AGJ, Iverson JB, Bour R, Fritz U, Georges A, Shaffer HB, Van Dijk PP, TTWG-Turtle Taxonomy Working Group (2017). Turtles of the world: Annotated checklist and atlas of taxonomy, synonymy, distribution and conservation status (8th edition). Chelonian Research Monograph.

[ref-27] Rovatsos M, Altmanová M, Johnson Pokorná M, Augstenová B, Kratochvíl L (2017a). Cytogenetics of the Javan file snake (*Acrochordus javanicus*) and the evolution of snake sex chromosomes. Journal of Zoological Systematics and Evolutionary Research.

[ref-28] Rovatsos M, Pokorná M, Altmanová M, Kratochvíl L (2014). Cretaceous park of sex determination: sex chromosomes are conserved across iguanas. Biology Letters.

[ref-29] Rovatsos M, Johnson Pokorná M, Altmanová M, Kratochvíl L (2015a). Female heterogamety in Madagascar chameleons (Squamata: Chamaeleonidae: *Furcifer*): differentiation of sex and neo-sex chromosomes. Scientific Reports.

[ref-30] Rovatsos M, Johnson Pokorná M, Altmanová M, Kratochvíl L (2016a). Mixed-up sex chromosomes: identification of sex chromosomes in the X_1_X_1_X_2_X_2_/X_1_X_2_Y system of the legless lizards of the genus *Lialis* (Squamata: Gekkota: Pygopodidae). Cytogenetic and Genome Research.

[ref-31] Rovatsos M, Johnson Pokorná M, Kratochvíl L (2015b). Differentiation of sex chromosomes and karyotype characterisation in the dragonsnake *Xenodermus javanicus* (Squamata: Xenodermatidae). Cytogenetic and Genome Research.

[ref-32] Rovatsos M, Praschag P, Fritz U, Kratochvil L (2017b). Stable Cretaceous sex chromosomes enable molecular sexing in softshell turtles (Testudines: Trionychidae). Scientific Reports.

[ref-33] Rovatsos M, Vukić J, Altmanová M, Johnson Pokorná M, Moravec J, Kratochvíl L (2016b). Conservation of sex chromosomes in lacertid lizards. Molecular Ecology.

[ref-34] Rovatsos M, Vukić J, Lymberakis P, Kratochvíl L (2015c). Evolutionary stability of sex chromosomes in snakes. Proceedings of the Royal Society B: Biological Sciences.

[ref-35] Sharma GP, Kaur P, Nakhasi U, Tiwari KK, Srivistava CB (1975). Female heterogamety in the Indian cryptodiran chelonian, *Kachuga smithi* Gray. Dr. B. S. Chuahah Commemoration Volume.

[ref-36] Spinks PQ, Shaffer HB, Iverson JB, McCord WP (2004). Phylogenetic hypotheses for the turtle family Geoemydidae. Molecular Phylogenetics and Evolution.

[ref-37] Sumner AT (1972). A simple technique for demonstrating centromeric heterochromatin. Experimental Cell Research.

[ref-38] Valenzuela N, Adams DC (2011). Chromosome number and sex determination coevolve in turtles. Evolution.

[ref-39] Yasukawa Y, Ota H, Hikida H (1992). Taxonomic re-evaluation of the two subspecies of *Geoemyda spengleri spengleri* (Gmelin, 1789) (Reptilia: Emydidae). Japanese Journal of Herpetology.

