# Supplementary material for: Turtles of the genera Geoemyda and Pangshura (Testudines: Geoemydidae) lack differentiated sex chromosomes: the end of a 40-year error cascade for Pangshura
Source: PeerJ. 2019 Feb 6;7:e6241. doi: 10.7717/peerj.6241 (PMC6368832; doi:10.7717/peerj.6241)
Supplement: Supplemental Information 1 [file peerj-07-6241-s001.docx]

>Geoemyda_japonica_cytb_Genbank_accession_number_MK097237

TGAAGTATAAGGTGGAGGCTGTTTGGCCAATAATGATGAATGGGTTTTCGACTGGTTGTCCTCCGATTCACGTAAGTACTAGTAGATCAGCTGTTAAGCATCAGAATAAGGTTTGGGTGAATGGTCGGAATAGGGCAGTACGTTGTTTTGACGTGTGCAGGATTGGTATAATAAATAGTACGAGGATGGAGGATAAAAGGGCAAGTACGCCTCCCAGTTTGTTTGGAATAGATCGTAGGATTGCGTAGGCAAATAGAAAATATCACTCCGGCTTAATGTGTGGAGGGGTGGATAGGGGGTTAGCTGGTGTAAAATTGTCTGGGTCGCCTAGAAGGTTAGGAAGGAATAGTGTTAGGCTTAGTAGGAGGGTTAGTATCAAGATGAGGCCTAGTAGATCTTTGTAGGAGAAATACGGGTGAAACGGGATTTTGTCCGTGTTTGAGTTTAACCCTGTTGGGTTGTTTGATCCGGTTTCATGGAGGAAGAGAAGGTGTACGGCTGCTAGACCGGCGATGGTGAAGGGTAGTAGGAAATGGAAAGTGAAGAATCGGGTTAAGGTGGCGTTGTCTACTGAAAATCCCCCTCAGATTCATTGTACCAAGGTATTACCGATATAAGGGGTGGCTGAGAGTAGGTTAGTGATGACGGTGGCGCCTCAGAATGATATTTGGCCTCAGGGTAAGACGTAGCCTATGAATGCAGTGGCTATGGTTAGAAGTAGTAGGATGATTCCTGTATTTCAGGTTTCTTTGTATAAGTATGAGCCGTAGTAAAGTCCTCGGCCAATGTGAAGGTAGATACATATAAAGAAGATGGAGGCTCCGTTGGCATGTGTATTGCGAATAAGTCATCCGTATTGCACATCTCGAGTAATATGGGCAACTGATGAGAATGCTATTGAGATATTAGGTGAGTAGTGTATTGCTAGGAAAATTCCAGTGATGATTTGTAGGATTAGGCAGGCGGCCAGGAGTGACCCGAAATTTCATCAAGCGGAGATGTTGGAGGGGCTTG

>Geoemyda_spengleri_cytb_Genbank_accession_number_MK097238

TTTTGTTTTCGATTAGTCCTGTGAGGGGTATTAGTACTAGGATAATTGTGAAGTACAGGGTAGAGGCTGCTTGGCCAATTATGATAAATGGGTTTTCGATTGGTTGTCCCCCGATTCATGTGAGTACCAGTAGATTAGCTGTTAGGCATCAGAATAGGGTTTGGGTTAGTGGTCGGAATAGGGCTGTTCGTTGTTTTGATGTATGTAGGGTTGGTATAATAAATAGTACAAGGATGGAAGATAATAGGGCAAGTACGCCACCCAGTTTATTTGGGATGGATCGTAGGATTGCATAGGCAAATAGGAAGTATCACTCTGGCTTAATATGCGGAGGAGTGGATAGTGGGTTGGCTGGTGTAAAATTGTCTGGGTCGCTCAATAGATTTGGAATGAATAGTGCTAGGCTTAGTAGGAGAGTTAGTATTAAGATAAGGCCTAGTAGATCTTTGTATGAGAAATATGGGTGGAATGGGATTTTGTCTGTGTTTGAGTTTAATCCTGTCGGGTTGTTTGATCCGGTTTCATGGAGGAAGAGAAGGTGTACGATTGCTAAGCCGGCGATGGTAAAAGGTAGTAGGAAATGGAAGGTGAAGAATCGGGTTAAGGTGGCGCCATCTACTGAAAATCCACCTCAAATTCATTGTACTAAAGTATTGCCAATATAGGGGATGGCTGAGAGTAGGTTGGTAATGACGGTAGCACCTCAGAATGATATTTGGCCTCAGGGTAAAACGTAGCCTATAAATGCAGTGGCTATAGTTAGAAATAGTAGGATGATTCCTGTGTTTCAGGTTTCTTTGTATAAATATGAGCCGTAGTAGAGTCCTCGGCCAATATGGATGTAGATGCATATGAAGAAGATGGAGGCCCCGTTGGCATGTGTGTTGCGAATAAGTCATCCGTATTGTACATCTCGAGTGATATGGGCAACTGATGAGAATGCTTGTGAGATGTCAGGTGAGTAGTGTATAGCTAGGAAAACTCCAGTGGTAATTTGTAGGATTAGGCAGGTGCCCAGGAGTGATCCGAAGTTTCATCAGGCGGAGATATTGGAGGGGCTTGGAAGATCAA

>Geoemyda_spengleri_cytb_Genbank_accession_number_MK097239

TTTTGTTTTCGATTAGTCCTGTGAGGGGTATTAGTACTAGGATAATTGTGAAGTACAGGGTAGAGGCTGCTTGGCCAATTATGATAAATGGGTTTTCGATTGGTTGTCCCCCGATTCATGTGAGTACCAGTAGATTAGCTGTTAGGCATCAGAATAGGGTTTGGGTTAGTGGTCGGAATAGGGCTGTTCGTTGTTTTGATGTATGTAGGGTTGGTATAATAAATAGTACAAGGATGGAAGATAATAGGGCAAGTACGCCACCCAGTTTATTTGGGATGGATCGTAGGATTGCATAGGCAAATAGGAAGTATCACTCTGGCTTAATATGCGGAGGAGTGGATAGTGGGTTGGCTGGTGTAAAATTGTCTGGGTCGCTCAATAGATTTGGAAGGAATAGTGCTAGGCTTAGTAGGAGAGTTAGTATTAAGATAAGGCCTAGTAGATCTTTGTATGAGAAATATGGGTGGAATGGGATTTTGTCTGTGTTTGAGTTTAATCCTGTCGGGTTGTTTGATCCGGTTTCATGGAGGAAGAGAAGGTGTACGATTGCTAAGCCGGCGATGGTAAAGGGTAGTAGGAAATGGAAGGTGAAGAATCGGGTTAAGGTGGCGCCATCTACTGAAAATCCACCTCAAATTCATTGTACTAAAGTATTGCCAATATAGGGGATGGCTGAGAGTAGGTTGGTAATGACGGTAGCACCTCAGAATGATATTTGGCCTCAGGGTAAAACGTAGCCTATAAATGCAGTGGCTATAGTTAGAAATAGTAGGATGATTCCTGTGTTTCAGGTTTCTTTGTATAAATATGAGCCGTAGTAGAGTCCTCGGCCAATATGGATGTAGATGCATATGAAGAAGATGGAGGCCCCATTGGCATGTGTGTTGCGAATAAGTCATCCGTATTGTACATCTCGAGTGATATGGGCAACTGATGAAAATGCTTGTGAGATGTCAGGTGAGTAGTGTATAGCTAGGAAAACTCCAGTGGTAATTTGTAGGATTAGGCAGGTGCCCAGGAGTGATCCGAAGTTTCATCAGGCGGAGATATTGGAGGGGCTTGGAAGATCAA

>Pangshura_smithi_cytb_Genbank_accession_number_MK097240

TCACCCGAGATGTCCAATACGGATGGCTTATCCGTAATATACATGCTAATGGGGCCTCCATCTTCTTCATATGCATCTACCTCCACATCGGCCGAGGTCTTTACTATAGCTCATACTTATACAAAGAAACCTGAAACACAGGAATCACTCTCTTATTCCTAACCATAGCCACCGCATTCGTAGGCTACGTCTTACCATGGGGCCAAATATCATTTTGAGGCGCTACTGTTATTACTAATCTACTCTCAGCCATCCCTTACATCGGCAACACCCTGGTACAATGAATCTGGGGCGGGTTCTCAGTAGATAACGCTACCCTGACCCGATTCTTCACCTTCCACTTCCTACTCCCCTTCACCATCGCCGGCCTAGCAACCGTCCACCTACTTTTCCTCCACGAAACCGGATCAAACAACCCTACAGGATTGAACTCAAACGCTGATAAAATCCCATTTCACCCCTACTTCTCATACAAAGACCTATTGGGCCTCATTCTTATACTAACCCTATTACTAAGTCTAGCACTATTCTTACCAAACCTCCTAACTGACCCGGACAACTTTACCCCCGCTAATCCCCTATCCACTCCCCCCCACATCAAACCAGAGTGATACTTCCTATTTGCCTACGCAATCCTCCGATCCATTCCCAACAAATTAGGGGGAGTACTAGCTCTACTACTCTCCGTCCTTGTATTATTCGCAATACCAACCCTACACACATCAAAACAACGCTCTACCCTATT
